# Tocilizumab for the Treatment of Rheumatoid Arthritis and Other Systemic Autoimmune Diseases: Current Perspectives and Future Directions

**DOI:** 10.1155/2012/946048

**Published:** 2012-01-18

**Authors:** Atsushi Ogata, Toshio Tanaka

**Affiliations:** Department of Respiratory Medicine, Allergy and Rheumatic Diseases, Osaka University Graduate School of Medicine, Suita City, Osaka 565-0871, Japan

## Abstract

Interleukin (IL)-6 is a cytokine featuring redundancy and pleiotropic activity. While IL-6, when transiently produced, contributes to host defense against acute environmental stress, continuous dysregulated IL-6 production plays a significant pathological role in several systemic autoimmune diseases. In response to the expectation that IL-6 blockade would constitute a novel therapeutic strategy for the treatment of these diseases, tocilizumab, a humanized anti-IL-6 receptor antibody, was developed. Clinical trials have verified the efficacy and the safety of tocilizumab for patients with rheumatoid arthritis, resulting in approval of this innovative biologic for the treatment of rheumatoid arthritis in more than 90 countries worldwide. Pathological analyses of the effect of IL-6 on the development of autoimmune diseases and a considerable number of case reports and pilot studies have also indicated the beneficial effects of this antibody on other systemic autoimmune diseases, including systemic lupus erythematosus, systemic sclerosis, polymyositis, and large-vessel vasculitis.

## 1. Introduction

Interleukin (IL)-6 is a cytokine featuring redundancy and pleiotropic activity. It was successfully cloned in 1996 as a B-cell differentiation factor, which promotes B-cell differentiation into antibody-producing cells [1]. Subsequent in vitro studies and analysis of IL-6 transgenic mice have shown that IL-6 acts not only on B cells but also on T cells, hepatocytes, hematopoietic progenitor cells, and various other cells [2–4]. One of the important functions of IL-6 is the differentiation of CD4^positive^ naïve T cells into effector cells. IL-6 in the presence of TGF-*β* promotes naïve T-cell differentiation into Th17 cells, while IL-6 inhibits TGF-*β*-induced regulatory T-cell (Treg) differentiation [5], causing imbalance between Th17 and Treg, which is a primary pathogenic factor in several autoimmune diseases [6].

IL-6 transmits its signal through its binding to transmembrane receptors or the soluble IL-6 receptor (IL-6R) [7, 8]. After binding of IL-6 to IL-6R, the resultant IL-6/IL-6R complex associates with gp130 and induces homodimerization of gp130, which triggers signal transduction system [9].

The pathological significance of IL-6 for diseases was first demonstrated in a case of cardiac myxoma [10]. The culture fluid obtained from the myxoma tissues of a patient who presented with fever, arthritis with positivity for antinuclear factor, increased C-reactive protein (CRP) levels and hypergammaglobulinemia and was diagnosed with undifferentiated connective tissue disease, contained a large quantity of IL-6, which suggested that IL-6 might contribute pathologically to chronic inflammation and autoimmunity. Subsequent studies have shown that dysregulation of IL-6 production is implicated in the pathogenesis of Castleman's disease [11], rheumatoid arthritis (RA) [12], and various other autoimmune, inflammatory, and malignant diseases [2–4].

Because of the biological activities of IL-6 and its pathological role in diseases, it was anticipated that IL-6 blockage would constitute a novel treatment strategy for autoimmune and inflammatory diseases [4, 13–15]. To this end, tocilizumab was developed, which is a humanized anti-IL-6R monoclonal antibody (Ab) of the IgG1 class that was generated by grafting the complementarity determining regions of a mouse anti-human IL-6R Ab onto human IgG1. Tocilizumab blocks IL-6-mediated signal transduction by inhibiting IL-6 binding to transmembrane and soluble IL-6R.

## 2. Approval of Tocilizumab for the Treatment of Rheumatoid Arthritis

### 2.1. Pathological Role of IL-6 in Rheumatoid Arthritis

RA is a chronic, progressive inflammatory disease of the joints and surrounding tissues accompanied by intense pain, irreversible joint destruction, and systemic complications such as fatigue, anemia, and fever [16]. At the local level, inflammatory cells invade the otherwise relatively acellular synovium leading to neovascularization, synoviocyte hyperplasia, and formation of pannus tissue, which in turn causes destruction of cartilage, erosion of the adjacent bone, and, ultimately, loss of function of the affected joint. The biological activities of IL-6 such as proinflammatory activity, augmentation of synovial fibroblast proliferation, osteoclast differentiation, matrix metalloproteinase (MMP), and vascular endothelial growth factor (VEGF) production, as well as lymphocyte differentiation and its elevation in both serum and synovial fluids of patients with RA [17–22] indicate that IL-6 is one of the key cytokines involved in the development of RA.

**Figure 1:**
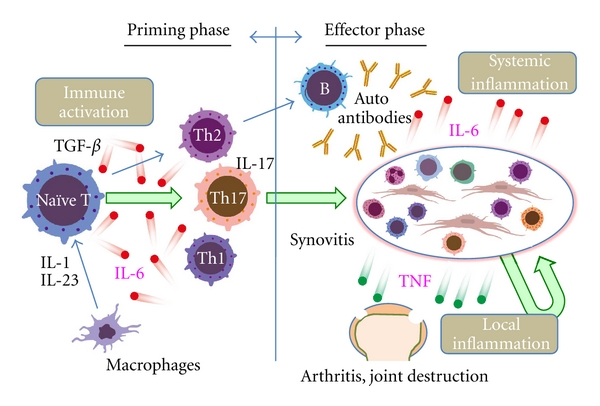
Pathological role of IL-6 in rheumatoid arthritis. IL-6 is important for development of Th17 and induction of autoantibodies such as rheumatoid factor. Activated Th17 cells and autoantibodies generate pannus in combination with activated fibroblastic synoviocytes, macrophages, and lymphocytes. Inflamed synovitis such as pannus is a major source of inflammatory cytokines including IL-6, and systemic inflammation (resulting in production of acute phase protein, anemia, and fatigue) is mainly mediated by IL-6. Tumor necrosis factor (TNF) plays a major role in the progression of local types of inflammation (arthritis) such as arthralgia, swelling, and joint destruction but plays a minor role during the priming phase.

It has been demonstrated in animal model of RA, that are type II collagen-induced arthritis (CIA), and antigen-induced arthritis, IL-6 performs a major role in the development and progression of joint destruction, while IL-6 deficiency generated by gene knockout or IL-6 blockade by means of anti-IL-6R Ab reduces the incidence and severity of arthritis in these models [23–28]. In the CIA model, immunization with type II collagen predominantly increased the frequency of Th17 cells and treatment of mice with anti-IL-6R Ab during priming markedly suppressed the induction of Th17 cells and arthritis development, while treatment with anti-IL-6R Ab on day 14 failed to suppress both Th17 differentiation and arthritis [29]. Similarly, in a glucose-6-phosphate-isomerase- (GPI-)induced arthritis model, administration of anti-IL-6R Ab on day 0 or 3 suppressed Th17 differentiation and protected against arthritis induction, while injection of anti-IL-6R Ab on day 14, at the peak of arthritis, did not bring about any improvement in arthritis [30]. Arthritis of anti-type II collagen antibody-induced arthritis (CAIA) is another arthritis model, but, in this model, the priming phase of T cell dependent antibody generation is skipped. Although IL-6 is also elevated in this model, CAIA was profoundly suppressed in TNF^−/−^ mice but not in IL-6^−/−^ mice [31], indicating that TNF may play a more significant role in the development of CAIA than IL-6. These observations suggest that in the priming phase IL-6 is a required factor for the activation of T cell response and production of antibodies specific for joint components and that in the effector phase TNF is the main generator of arthritis [32]. We found that tocilizumab was not effective for clinical improvement in the condition of two patients with psoriatic arthritis, for whose development immune activation does not appear to be required [33]. The clinical antiarthritic effect of tocilizumab is slower than that of TNF inhibitors, which may be due to the different pathological roles of IL-6 and TNF in the development of RA (Figure 1).

### 2.2. Efficacy of Tocilizumab in Randomized Controlled Trials

As shown in Table 1, seven phase III clinical trials of tocilizumab subsequent to phase I and II studies demonstrated its efficacy either as monotherapy or in combination with disease-modifying antirheumatic drugs (DMARDs) for adult patients with moderate to severe RA [34–40]. A Cochrane database systematic review concluded that tocilizumab-treated patients taking concomitant methotrexate were four times more likely to achieve American College of Rheumatology (ACR) 50 improvement (absolute %, 38.8% versus 9.6%) and 11 times more likely to achieve Disease Activity Score (DAS) remission (30.5% versus 2.7%) than patients taking a placebo [41]. Furthermore, the SAMURAI [34] and LITHE studies [40] proved that radiological damage of joints was significantly inhibited by the treatment. The findings of the RADIATE trial showed that, among RA patients who had previously discontinued TNF inhibitors 50% achieved ACR20, 28.8% ACR50, and 12.4% ACR70 responses [36]. The ACR improvement and DAS remission criteria include an acute-phase reactant component, so that there was concern that the effect of tocilizumab evaluated with these criteria might be overestimated. However, it was found that, even when criteria such as the Simplified Disease Activity Index (SDAI) and Clinical Disease Activity Index (CDAI) were used, remission rates for patients treated with tocilizumab were in the same range as those for patients treated with TNF inhibitors [42, 43].

### 2.3. Efficacy of Tocilizumab in Actual Medical Practice

On the basis of the excellent results obtained for the efficacy of tocilizumab, it was approved in April 2008 for the treatment of RA in Japan. The recommended posology of tocilizumab (proprietary name, Actemra) is 8 mg/kg, every 4 weeks. Subsequently, the European Medicines Agency approved tocilizumab (proprietary name, RoACTEMRA) for RA in January 2009 at a recommended dose of 8 mg/kg. In the United States, it was approved for RA in January 2010, but the recommended starting dose is 4 mg/kg administered once every 4 weeks followed by an increase to 8 mg/kg depending on clinical response. While the dosage differs among countries, tocilizumab has now been approved for the treatment of RA in more than 90 countries worldwide [14].

In addition to clinical trials, the efficacy of tocilizumab was reconfirmed in actual medical practice. The finding by the three recent studies, the German phase IIIb real-life study (TAMARA study) [44, 45], the Danish nationwide cohorts of RA patients (DANBIO registry) study [46], and the multicenter retrospective real-life study (REACTION study) [47, 48] are shown in Table 2. In the TAMARA study, 286 patients were registered for an analysis of the effectiveness and safety [44, 45], 41.6% of whom had previously been treated with TNF inhibitors. ACR50 and ACR70 response rates at week 24 were 50.7% and 33.9%, respectively, while 47.6% of the patients achieved DAS remission and 54.9% the European League Against Rheumatism (EULAR) good response. Remission rates with the new ACR/EULAR Boolean-based criteria for clinical studies were 15.0% after 12 weeks and 20.3% after 24 weeks, and CDAI and SDAI remission rates were 24.1% and 25.2%, respectively. For the DANBIO registry in Denmark, 178 patients with RA treated with tocilizumab were identified [46]. The disease activity decreased at all-time points, with remission rates for tocilizumab treatment of 39% after 24 weeks and 58% after 48 weeks. EULAR good or moderate response rates were 88% and 84%, respectively. These response rates were comparable to those found for patients switching to their second TNF inhibitors and to the response rates previously observed in phase III clinical trials. In Japan, 229 patients were registered in the REACTION study for an analysis of the effectiveness of tocilizumab [47, 48]. Clinical remission at week 52 was observed in 43.7% of the patients, radiographic non-progression in 62.8%, and functional remission in 26.4%. The retention rates at 24 and 52 weeks were 79.5% and 71.1%, respectively, and were the same for those with or without previous anti-TNF treatment. These results indeed show the efficacy of tocilizumab for the treatment of RA in actual medical practice.

### 2.4. Safety Profile of Tocilizumab

The safety and tolerability profiles of tocilizumab monotherapy for Japanese RA patients obtained from six initial trials and five long-term extensions have been published [49]. For these studies, 601 patients with a total exposure to tocilizumab of 2,188 patient-years (pt-yr) were enrolled. The medial treatment duration was 3.8 years. The incidence of adverse events (AEs), including abnormal laboratory test findings, was calculated as 465/100 pt-yr, with infections being the most common serious AEs (6.2/100 pt-yr). Of the patients treated more than 5 years, 59.7% met the DAS28 remission criteria at 5 years, which demonstrates the excellent tolerability and high efficacy of tocilizumab. In addition, a systemic literature review to assess the risk of AEs for RA patients treated with tocilizumab reported that pooled odds ratios (ORs) indicated statistical significance for an increased risk of AEs for patients treated with 8 mg/kg of tocilizumab plus methotrexate compared with controls (OR = 1.53; 95%CI = 1.26–1.86), as well as a heightened risk of infection (OR = 1.30; 95%CI = 1.07–1.58) [50]. However, no increases in the incidence of malignancy or hepatitis were detected.

The results of an interim analysis of a postmarketing surveillance of all patients treated with tocilizumab in Japan were recently reported [51]. This analysis comprised 3,881 patients who received 8 mg/kg of tocilizumab every 4 weeks, and was observed for 28 weeks. Occurrence of a total of 3,004 AEs in 1,641 patients (167/100 pt-yr) and 490 serious AEs in 361 patients (27/100 pt-yr) was reported. The most frequent AE and serious AE were infection at 31/100 pt-yr and 9/100 pt-yr, respectively, with the majority of infections being pneumonia and cellulitis. Cardiovascular events were observed in 0.9% (myocardial infarction in 4 patients or 0.1%). Abnormalities in laboratory test findings, such as increases in lipid and liver function parameters were common, and total and serious AEs associated with laboratory test abnormalities were 35/100 pt-yr and 2/100 pt-yr, respectively. The increased lipid level resulting from tocilizumab administration is perhaps mediated by its effecting on lipoprotein receptor expression, since it was recently shown that overproduction of IL-6 reduces blood lipid levels via upregulation of very-low-density lipoprotein receptors [52]. In contrast, we and others observed that HbA1c levels and insulin sensitivity improved as a result of tocilizumab treatment [53, 54]. While white blood cell and neutrophil counts usually decreased just after tocilizumab injection, this was not related to the incidence of infection. Twenty-five patients died for a standardized mortality ratio of 1.66, which was similar to the results reported for a Japanese cohort study of RA. The results of this analysis thus demonstrated that tocilizumab is acceptable in the actual clinical setting.

Seven cases of gastrointestinal (GI) perforation in six patients were reported in this postmarketing surveillance. In the worldwide Roche clinical trials, 26 (0.65%) cases of GI perforation were found among patients with RA treated with tocilizumab for a rate of 1.9/1,000 pt-yr and most cases appeared to be complications of diverticulitis [55]. This rate is intermediate between the rates of GI perforations of 3.9/1,000 pt-yr for corticosteroids and 1.3/1,000 pt-yr for anti-TNF*α* agents reported in the United Health Care database.

The reactivation of tuberculosis is a major concern during anti-TNF treatment [56], but there is no medical consensus regarding the effect of IL-6 blockade on tuberculosis. Okada et al. examined the effects of IL-6 and TNF*α* blockade on the development of tuberculosis infection in mice and observed that there was less tuberculosis infection for anti-IL-6R Ab than for anti-TNF*α* Ab [57]. In addition, we showed that tuberculosis antigens-induced interferon (IFN)-*γ* production was suppressed by the addition of TNF inhibitors (infliximab and etanercept) but not of tocilizumab [58]. Although it seems likely that the incidence of reactivation of tuberculosis is lower during tocilizumab treatment than that during anti-TNF treatment, further detailed studies will be needed to clarify this point.

### 2.5. The Place of Tocilizumab in Rheumatoid Arthritis Treatment

A number of biologics are available for the treatment of RA. These include anti-TNF blockers (infliximab, etanercept, adalimumab, golimumab, and certolizumab), an IL-1 antagonist (anakinra), a B-cell depletor (rituximab), an IL-6 receptor inhibitor (tocilizumab), and a T-cell activation blocker (abatacept). These biological modifiers target different molecules and B cells, leading to different clinical effects and causing different adverse effects. Since no head-to-head comparative studies have been made of the efficacy of these various agents, it has not yet been determined which of these biologics should be selected for a given patient. Currently, one of the anti-TNF drugs is chosen as a first-line biologic, but between 14 and 38% of patients show no or little response to anti-TNF treatment, with as many as 40% of patients discontinuing these drugs within a year and 50% within 2 years. The findings of the RADIATE trial showed that RA patients who had previously discontinued TNF inhibitors, mainly due to their inefficacy, achieved ACR20/50/70 responses of 50%, 28.8%, and 12.4%, respectively, when tocilizumab was administered at 8 mg/kg every four weeks [36]. At present, tocilizumab is likely to be prescribed as a second-line biologic therapy but will have to overcome significant competition from established anti-TNF therapies.

It is anticipated that tocilizumab will be selected as a first-line biologic for moderately to severely active RA patients with certain complications. AA amyloidosis is a serious complication of RA, and amyloid fibril deposition causes progressive deterioration in various organs [59, 60]. Since the gene activation of serum amyloid A, a precursor protein of amyloid A fibril, depends primarily on IL-6 [61, 62], tocilizumab administration was found to promptly reduce serum concentrations of SAA, just as in the case of CRP [60]. Three case reports showed the clinical ameliorative effect of tocilizumab on gastrointestinal symptoms due to intestinal amyloidosis [63–65], and amyloid A fibril deposits were found to have disappeared in two cases after three injections of tocilizumab [63, 65]. This suggests that tocilizumab may be suitable as a first-line drug for RA patients who are complicated with or are at high risk of developing AA amyloidosis.

### 2.6. Drug-Free Remission Rate

Remission induction is the current goal for RA, and with the development of biological modifiers, a growing number of RA patients has been able to achieve this goal [66]. The long-term efficacy after cessation of tocilizumab followed by DAS28 remission was demonstrated in the DREAM (drug-free remission after cessation of actemra monotherapy) study [67]. The continuous rate of tocilizumab-free efficacy was 35.1% at 24 weeks and 13.4% at 52 weeks. Serum levels of IL-6 and MMP-3 are useful markers for identifying patients who may be able to discontinue tocilizumab without risk of recurrence. In addition, the RESTORE study (retreatment efficacy and safety to tocilizumab in patients with rheumatoid arthritis at recurrence) demonstrated that retreatment of all relapsed patients with tocilizumab resulted in re-remission [68].

## 3. Therapeutic Implications of Tocilizumab for Other Systemic Autoimmune Diseases

### 3.1. Systemic Lupus Erythematosus

Systemic lupus erythematosus (SLE) is a multisystem autoimmune disorder with a broad spectrum of clinical presentations of unknown etiology that mainly affects young women [69]. The pathogenesis of SLE remains unclear, but the concept of apoptosis goes some way towards explaining how the immune system may recognize mainly intracellular antigens. Defects in the clearance of apoptotic cells have been recognized in SLE patients, leading to aberrant uptake by macrophages, which then present intracellular antigens to T and B cells, thus driving the autoimmune process [70]. Cytokine dysregulation is pervasive, and its expression profiles may serve as a marker of disease activity and severity. Recent findings have highlighted type I interferon pathway [71] or Th17 cell activation [72] in the pathogenesis of SLE.

Levels of CRP have been shown to rise in acute illness but not in SLE flares, indicating that IL-6, a major regulator of CRP production, has a minor role in SLE development. However, recent findings suggest that CRP dysregulation also plays a part in the pathogenesis of SLE [73] and SLE may well be a potential target for IL-6 blockade [74]. Serum IL-6 levels of SLE patients were elevated [75–77]. Urinary excretion and renal expression of IL-6 was elevated in SLE patients with active proliferating lupus nephritis [76, 78–81], as were IL-6 levels in the cerebrospinal fluid of SLE patients with central nervous system involvement [82]. Compared to healthy controls, SLE patients had significantly more IL-6 secreting peripheral blood mononuclear cells [83, 84]. Lymphoblastoid cells isolated from SLE patients produced higher levels of IL-6 and blocking of IL-6 inhibited anti-double-stranded DNA (dsDNA) Ab production in vitro [85, 86], indicating that IL-6 is involved in autoantibody production. In murine SLE models, age-associated increases in serum IL-6, soluble IL-6R, and abnormal expression of IL-6R have been detected in MRL/lpr mice [87–89]. In old NZB/W mice, anti-IL-6 Ab reduced and exogenous IL-6 increased production of IgG dsDNA Ab by B cells [90, 91]. Furthermore, IL-6 administration exacerbated glomerulonephritis [92, 93], while IL-6 blockade by means of anti-IL-6R or anti-IL-6 Ab prevented the onset and progression of the disease [94, 95]. Mice with epidermal loss of JunB reportedly developed an SLE phenotype linked to increased epidermal IL-6 secretion, and facial skin biopsies of SLE patients displayed low levels of JunB protein expression, high IL-6, and activated STAT3 levels within lupus lesions [96]. These findings led to an open-label phase I dosage-escalation study of tocilizumab (2 mg/kg, 4 mg/kg or 8 mg/kg, every 2 weeks for 12 weeks) with an enrollment of 16 SLE patients with mild-to-moderate disease activity [97]. Significant improvement in the modified Safety of Estrogens in Lupus Erythematosus National Assessment version of the Systemic Lupus Erythematosus Disease Activity Index score was observed in 8 of the 15 evaluable patients, accompanied by a median reduction in anti-dsDNA Ab levels of 47%. The percentage of CD38^high^CD19^low^IgD^negative^ plasma cells in the peripheral blood, which was higher for SLE patients than for normal controls (mean 5.3% versus 1.2%), was significantly reduced to 3.1% at 6 weeks. These results indicate that tocilizumab represents a promising therapeutic biologic for SLE.

### 3.2. Systemic Sclerosis

Systemic sclerosis (SSc) is a connective tissue disease, characterized by fibrosis of the skin and internal organs, vasculopathy, and immune abnormalities [98]. IL-6 is a definite therapeutic target in SSc [99]. IL-6 in the serum of SSc patients was reportedly elevated and the level correlated with the skin severity score [100–104]. Moreover, the culture supernatants of peripheral blood mononuclear cells and skin tissues from SSc patients contained higher concentrations of IL-6 than those from controls [105–109]. In vitro studies demonstrated that IL-6 may contribute to fibrosis by inducing collagen production [110] and induce *α*-smooth muscle actin (*α*-SMA) expression by dermal fibroblasts [111], leading to their differentiation into myofibroblasts. On the other hand, anti-IL-6 Ab suppressed procollagen type 1 production in fibroblasts derived from SSc patients in vitro [112]. SSc serum mediated largely by IL-6 was found to induce endothelial cell activation and apoptosis in endothelial cell-neutrophil cocultures [113]. IL-6 is also associated with humoral and cellular immunological abnormalities in SSc [98, 99]. IL-6 is thus thought to play a significant role in producing the characteristics of SSc. Moreover, in a SSc model mouse, induced by immunization with topoisomerase I and complete Freund's adjuvant, loss of IL-6 expression could ameliorate skin and lung fibrosis [114]. We also examined the clinical effect of tocilizumab on two diffuse SSc patients who had been resistant to conventional treatment regimens [115]. Six months after the treatment, both patients showed softening of the skin with reductions of 50.7% and 55.7% for the total *z*-score determined with the Vesmeter, a novel device for measuring the physical properties of the skin [116], and of 51.9% and 23% for the modified Rodnan total skin score. Histological examination showed thinning of the collagen fiber bundles and reduction of the number of *α*-SMA positive cells in the dermis. Since there are few therapeutic drugs for SSc at the present time [117], these improvements suggest that tocilizumab appears to be a promising biologic for the treatment of SSc.

### 3.3. Polymyositis

The inflammatory myopathies encompass a group of heterogenous muscle diseases which share the common clinical features of slowly progressive symmetrical muscle weakness, decreased muscle endurance, and fatigue [118]. They include polymyositis (PM), dermatomyositis, and inclusion body myositis, but are generally considered to be distinct diseases with different pathophysiological mechanisms. Muscles produce IL-6 [119], and IL-6 has been also shown to play a regulatory role in muscle wasting [120]. Among these inflammatory myopathies, PM appears to be another suitable target disease for tocilizumab. Excessive IL-6 expression has been found in the sera and infiltrating mononuclear cells in the muscles of PM patients [121–123]. Infiltrating cytotoxic T cells are thought to be involved in muscle fiber damage, and IL-6 functions as a helper factor in the induction of cytotoxic T cells [124]. Moreover, in a model of myosin-induced experimental myositis it was shown that control mice developed clinically manifest muscle damage, whereas IL-6-deficient mice showed no clinical or histological signs of muscle damage [125]. In another model of PM, known as C-protein-induced myositis, intraperitoneal administration of anti-IL-6R Ab suppressed the severity of myositis preventatively as well as therapeutically [126]. We tested the efficacy of tocilizumab in two PM patients who had been refractory to corticosteroids and immunosuppressive drugs [127]. Creatine phosphokinase levels of both patients normalized and MR images showed the disappearance of high-intensity zones in the thigh muscles. These findings suggest that tocilizumab may also be effective as a novel drug for refractory PM.

Dermatomyositis is a complement-mediated microangiopathy associated with destruction of capillaries, hypoperfusion, and inflammatory stress on the perifascicular regions, so that the pathology is different from that of PM [118]. Production of IL-6 and type I interferon signature genes was recently proposed as a biomarker for disease activity in childhood dermatomyositis [128], which thus may be another disorder suitable for tocilizumab targeting.

### 3.4. Takayasu's Arteritis and Giant Cell Arteritis

Vasculitis refers to inflammation where blood vessels are the primary site of inflammation. The pathological consequence of such inflammation is destruction of the vessel wall, which is histologically detected as fibrinoid necrosis. Takayasu's arteritis (TA) and giant cell arteritis (GCA) belong to an entity designated vasculitis syndrome, and involve both large and medium-sized arteries [129, 130]. The pathogenesis of TA and GCA remains unclear, but it is clear that IL-6 is involved in their development [129–133]. Tocilizumab treatment for a 20-year-old woman with refractory active TA improved the clinical manifestations and abnormal laboratory findings [134], and subsequent studies reported that tocilizumab treatment induced a rapid remission in 2 patients with TA and 5 patients with GCA [135]. Surprisingly, two of the patients with GCA went into remission without concomitant use of corticosteroids. Moreover, tocilizumab was also shown to be effective as rescue treatment for three GCA patients for whom the prednisone dose could not be tapered to less than 30 mg/day [136]. Positron emission tomography/CT scans revealed that in two patients generalized large-vessel vasculitis was detected during the active phase, which completely resolved upon a 6-month course of tocilizumab therapy. These reports strongly imply that IL-6 inhibition may serve as an innovative strategy for the treatment of both TA and GCA. However, several studies have suggested that GCA patients with a lesser inflammatory response without an increase in IL-6 expression were at a higher risk of developing ischemic manifestations than were other patients [137], since the angiogenic activity of IL-6 offers protection against ischemia in such GCA patients [138]. These findings indicate that further clinical studies are required to evaluate the efficacy and safety of tocilizumab for GCA and TA.

It is worthy of note that IL-6 has been also implicated in the development of other types of vasculitis syndrome such as polyarteritis nodosa (PAN) and antineutrophil-cytoplasmic-antibody- (ANCA) associated vasculitis [139–142]. However, so far there have been no reports about off-label use of tocilizumab for PAN or ANCA-associated vasculitis.

## 4. Therapeutic Implications for Other Autoimmune and Inflammatory Diseases

On the basis of excellent results of the efficacy of tocilizumab for Castleman's disease [143, 144] and systemic juvenile idiopathic arthritis [145–147], it has been approved and used as the first-line biologic in Japan. Pilot studies and case reports with off-label use of tocilizumab also indicate the potential indications of this biologic for various other organ-specific autoimmune and chronic inflammatory diseases. These include relapsing polychondritis [148], acquired hemophilia A [149], autoimmune hemolytic anemia [150], adult-onset Still's disease [151–165], Crohn's disease [166], Bechet's disease with posterior uveitis [167], polymyalgia rheumatica [135, 168], remitting seronegative, symmetrical synovitis with pitting edema [169], spondyloarthritides [170–175], graft-versus-host disease [176, 177], TNF-receptor-associated periodic syndrome [178], and pulmonary arterial hypertension complicated with Castleman's disease or mixed connective tissue disease [179–181]. Further clinical trials are essential, however, to evaluate the efficacy and safety of tocilizumab for these diseases.

## 5. Conclusion

Acute IL-6 synthesis provides a warning signal and protects the host from environmental stress, while its prolonged production causes the onset and progression of various autoimmune diseases. Several clinical trials have verified the efficacy and safety of tocilizumab for RA, systemic juvenile idiopathic arthritis and Castleman's disease, resulting in approval of this innovative biologic for the treatment of these diseases. Case reports of off-label use or pilot studies have also raised the possibility that tocilizumab could become the biological drug of choice for other systemic autoimmune diseases including SLE, systemic sclerosis, polymyositis and large vessel vasculitis. At present, the mechanisms through which tocilizumab exerts its clinical ameliorative effects on phenotypically different autoimmune diseases are not completely understood. IL-6 blockade may suppress autoantibody production or correct the imbalance of autoantigen-specific Th17 and/or Th1 versus Treg. Thus, clarification of the mechanisms as well as further clinical trials to evaluate the efficacy and safety of tocilizumab for these diseases are important issues.

##  Conflict of Interests

Toshio Tanaka declares no conflict of interests.

## Figures and Tables

**Table 1:** Phase III randomized controlled trials of tocilizumab for RA patients. Summary of the results of seven phase III randomized controlled trials of tocilizumab. DMARDs: disease modifying antirheumatic drugs, IR: inadequate response, TCZ: tocilizumab, anti-TNF: anti-tumor necrosis factor inhibitor, MTX: methotrexate.

Study	Reported year	Population	Week at evaluation	Treatment arms	Patient (*n*)	Response rates (%)			Remission rate (%)	Radiological progression		
						ACR20	ACR50	ACR70	DAS28<2.6	TSS: Total Sharp score	ES: Erosion score	JSNS: Joint space narrowing score
SAMURAI	2007	DMARDs IR	52 W	TCZ (8)	157	78	64	44	59	2.3	0.9	1.5
				DMARDs	145	34	13	6	3	6.1	3.2	2.9

TOWARD	2008	DMARDs IR	24 W	TCZ (8) + DMARDs	803	61	38	21	30			
				DMARDs	413	25	9	3	3			

RADIATE	2008	Anti-TNF IR	24 W	TCZ (4) + MTX	161	30	17	5	8			
				TCZ (8) + MTX	170	50	29	12	30			
				placebo + MTX	158	10	4	1	2			

OPTION	2008	MTX IR	24 W	TCZ (4) + MTX	186	48	31	12	13			
				TCZ (8) + MTX	191	59	44	22	27			
				placebo + MTX	189	26	11	2	1			

SATORI	2009	MTX IR	24 W	TCZ (8)	61	80	49	30	43			
				MTX	64	25	11	6	2			

AMBITION	2010	MTX, anti-TNF naïve	24 W	TCZ (8)	286	70	44	28	34			
				MTX	284	53	34	15	12			

LITHE	2011	MTX IR	52 W	TCZ (4) + MTX	394	47	29	16	30	0.34	0.21	0.13
				TCZ (8) + MTX	398	56	30	20	47	0.29	0.17	0.12
				MTX	393	25	10	4	8	1.13	0.71	0.42

**Table 2:** Reevaluation of antirheumatic effects of tocilizumab in actual medical practice. Summary of the contents of the three actual medical practice of tocilizumab for rheumatoid arthritis.

Study	Country	Patient number	Registry	Evaluation
TAMARA	Germany	286	Sep. 2008~Sep. 2009	Disease activity
				EULAR response
				ACR response
				Adverse events
				2011 ACR/EULAR remission

DAMBIO	Denmark	178	~April 2010	Disease activity
				EULAR response
				Drug survival

REACTION	Japan	229	April 2008~March 2009	Disease activity
				EULAR response
				Adverse events
				Drug survival
